# PD-L1 Immunohistochemistry Comparability and Their Correlation with Clinical Characteristics in NSCLC

**DOI:** 10.1155/2020/3286139

**Published:** 2020-11-02

**Authors:** Chiao-En Wu, Ching-Fu Chang, Liao Kou-Sheng, Ju Chiang, Shih-Wei Lee, Yu-Chi Chiu

**Affiliations:** ^1^Division of Haematology-Oncology, Department of Internal Medicine, Chang Gung Memorial Hospital at Linkou, Chang Gung University College of Medicine, Taoyuan, Taiwan; ^2^Department of Pathology, Taoyuan General Hospital, Ministry of Health and Welfare, Taoyuan, Taiwan; ^3^Department of Internal Medicine, Taoyuan General Hospital, Ministry of Health and Welfare, Taoyuan, Taiwan

## Abstract

**Background:**

PD-L1 expression is an important predictive factor of response to therapy with immune checkpoint inhibitors (ICIs). This study was designed to retrospectively analyze the concordance of PD-L1 measurements using three different assays (Dako22C3, Dako28-8, and SP142) in NSCLC patients and to find possible predictors of high PD-L1 expression.

**Materials and Methods:**

Data of 144 patients with histologically confirmed NSCLC and available PD-L1 measurements treated at the Taoyuan General Hospital from 2018 to 2019 were retrospectively reviewed in the study. Patients' characteristics, including age, sex, clinical stage (T, N, and M) of NSCLC (AJCC, 8^th^ edition), and EGFR/ALK alterations, were analyzed for association with PD-L1 expression.

**Results:**

Measurements of PD-L1 expression levels with Dako22C3 and Dako28-8 were comparable while SP142 showed lower levels of PD-L1 expression. The overall agreement between Dako22C3 and Dako28-8 was 82.2% and 91.6% for both 1% and 50% TPS cut-offs, respectively. The above findings were confirmed by Cohen's kappa. In addition, we found that PD-L1 expression was significantly associated with advanced N stage but not with T and M stages.

**Conclusion:**

Dako22C3 and Dako28-8 showed comparable results in assessing PD-L1 levels. Future prospective studies are needed to validate these findings. N stage may be a good predictor for PD-L1 expression.

## 1. Introduction

Since the discovery and understanding of Programmed Cell Death 1 (PD-1)/Programmed Cell Death Ligand 1 (PD-L1) interactions between cancer and immune cells, blocking the PD-1/PD-L1 interaction has become a novel therapeutic strategy in cancer treatment. Anti-PD-1/PD-L1 immune checkpoint inhibitors (ICIs), including pembrolizumab [1–4], nivolumab [5–7], and atezolizumab [8–10], alone or in combination with chemotherapy, have been approved for metastatic non-small cell lung cancer (NSCLC) as later-line [1, 5–9] and first-line treatments [2–4, 10].

PD-L1 is the major target of ICIs, and PD-L1 expression assessed using immunohistochemistry (IHC) is considered a predictive biomarker for response to ICIs in NSCLC [1, 3, 7, 9, 11, 12] and other cancers such as gastric cancer [13]. PD-L1 has been reported as a prognostic factor in cancer [14–16], but the prognostic value of PD-L1 is still controversial as some studies showed no significant correction between PD-L1 and survivals [17, 18]. PD-L1 IHC is evaluated by experienced pathologists and scored as the percentage of tumor cells with membrane staining of any intensity (the tumor proportion score, TC or TPS) and the percentage of immune cells with similar staining (the immune cell proportion, IC). Clinical trials of ICIs have evaluated the PD-L1 expression using different assays and antibodies, raising the question whether these assays could be interchangeable. Previous studies, including two prospective studies sponsored by the National Comprehensive Cancer Network and the Blueprint Project [19, 20], have compared the sensitivity and reproducibility of different assays for detecting PD-L1 expression [21]. In general, SP142 showed lower sensitivity than other FDA-approved assays such as Dako22C3 (22C3) and Dako28-8 (28-8).

To validate these findings in NSCLC patients with a high prevalence of EGFR mutations, we retrospectively analyzed the assay concordance of PD-L1 IHC staining in NSCLC patients and attempted to determine the possible predictors of high PD-L1 expression.

## 2. Materials and Methods

### 2.1. Patients and Characteristics

Data of 306 patients with histologically confirmed NSCLC, treated at Taoyuan General Hospital from 2018 to 2019, were retrospectively reviewed. Among them, 144 patients with available PD-L1 IHC data were included in the study. Patients' characteristics, including age, sex, clinical stage (T, N, and M) of NSCLC (AJCC, 8th edition), and EGFR/ALK alterations, were recorded. ALK was determined by IHC staining.

### 2.2. IHC Staining of PD-L1

Two to eight 5 *μ*m sections were cut from each patient at the Taoyuan General Hospital and sent to the Taipei Institute of Pathology for staining as follows: assay 1, 22C3 on the Dako Link 48 platform; assay 2, 28-8 on the Dako Link 48 platform; assay 3, SP142 on the VENTANA BenchMark platform.

The stained slides were scored by pathologists according to the scoring protocol of each system. The TPS was applied for 22C3 and 28-8, and TC/IC was applied for SP142. PD-L1-stained TCs were scored in terms of TPS, which represents the percentage of TC showing membranous PD-L1 staining, and they were also classified into one of the three categories (<1%, 1%–49%, and >50%). PD-L1-stained ICs were scored based on the scoring approach described in the VENTANA SP142 PD-L1 IHC assay unlike 22C3 and 28-8 which use TPS alone; SP142 scores both TC/IC and the assay is defined as “negative” if both TC/IC are lower to 1%, “high expression of PD-L1” if TC is higher to 50% or IC higher to 10%; all other scores are classified as “intermediate levels of PD-L1brochure” and classified into one of the three categories (<1%, 1%–9%, and >10%). In these assays, the score of the TPS or TC/IC is based on membrane and cytoplasmic staining of any intensity. Unlike 22C3 and 28-8 which use TPS alone, SP142 scores both TC/IC, and the assay is defined as “negative” if both TC/IC are lower to 1% and “high expression of PD-L1” if TC is higher to 50% or IC higher to 10%; all other scores are classified as “intermediate levels of PD-L1.”

### 2.3. Statistical Analysis

To assess the different results from different assays, the Fisher-Freeman-Halton test of independence was used for categorical variables. The agreement between assays was examined by Cohen's kappa coefficient (*κ*) [22] while the null hypothesis is when the agreement between assays is due to chance. A *p* value less than 0.05 was considered statistically significant. IBM SPSS Statistics for Windows (Version 20.0, Armonk, NY, USA) was used for statistical analyses. Venn diagrams were used to present the agreement between the assays. This study was approved by the Medical Ethics and Institutional Review Board of Taoyuan General Hospital, Ministry of Health and Welfare (TYGH 109022).

## 3. Results

### 3.1. Patient Characteristics

A total of 144 NSCLC patients, with available data of PD-L1 expression from at least one assay, were included in the study. The median age was 65 years, ranging from 28 to 94 years. Ninety-two patients (63.9%) were male and 52 patients (36.1%) were female. In terms of histology, most patients had adenocarcinoma (*n* = 101, 70.1%), 19 had squamous cell carcinoma (13.2%), 5 had adenosquamous carcinoma (3.5%), and 19 had NSCLC (13.2%), which were not classified in any of the previous types. Most patients (*n* = 120, 83.3%) had been diagnosed as stage IV. Fifty-nine (52.4%) of 124 patients had an EGFR mutation, and 6 (5.0%) of 121 had ALK alterations. Patients' characteristics are summarized in Table 1.

### 3.2. PD-L1 Expressions Using 22C3, 28-2, and SP142

**Figure 1:**
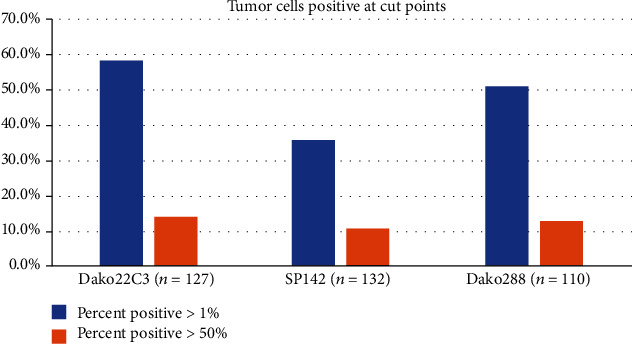
The proportion of PD-L1 expression assessed by 3 different assays.

Among 127 patients with PD-L1 using 22C3, 18 (14.2%) had TPS > 50% and 74 (58.3%) had TPS > 1%. Among 110 patients with PD-L1 using 28-8, 14 (12.7%) had TPS > 50% and 56 (51.9%) patients had TPS > 1%. In contrast, among 132 patients with PD-L1 using SP142, 14 (10.6%) had TC/IC > 50%/10% and 47 (35.6%) had TC/IC > 1%/1% (Table 1 and Figure 1), which were lower than PD-L1 using the other two assays.

### 3.3. Comparability of PD-L1 Expressions Using 22C3, 28-2, and SP142

PD-L1 expression levels, assessed with different assays, were compared, and the results are shown in Tables 2 and 3. When the cut-off was 1%, the overall agreement between 22C3 and 28-8 was 82.2%. However, overall agreement was 62.6% between SP142 and 22C3 and 62.6% between SP142 and 28-8, possibly resulting from SP142 having lower sensitivity than the other two assays when the cut-off was set at 1%. By using Cohen's kappa, similar trends were found as *κ* was higher between 22C3 and 28-8 (*κ* = 0.645) than SP142 and 22C3 (*κ* = 0.299) and SP142 and 28-8 (*κ* = 0.251). In contrast, at 50% cut-off, overall agreements were higher than 90% among the three assays, possibly due to the low proportion (10-15%) of patients expressing high PD-L1 levels. By using Cohen's kappa, all the paired assays have compatible *κ* values (*κ* = 0.565, 0.569, and 0.593 for SP142/28-8, SP142/22C3, 22C3/28-8, respectively).

**Figure 2:**
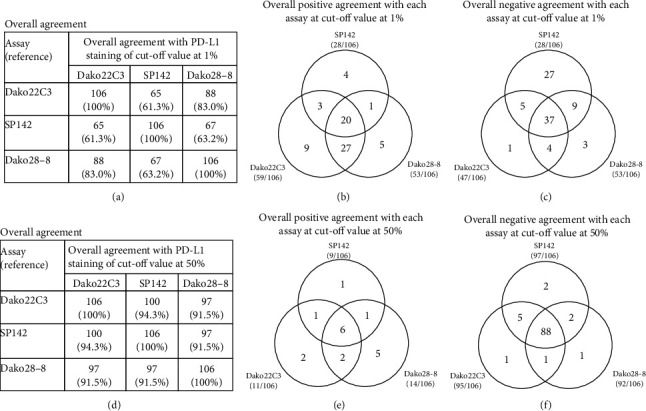
Overall agreement in 106 patients with PD-L1 using all three assays at a cut-off of 1% (a–c) and at a cut-off of 50% (d–f). Venn diagrams showing the positive (b, e) and negative agreement (c, f) at cut-offs of 1% (b, c) and 50% (e, f).

For 106 patients who had their PD-L1 levels assessed with all three assays, similar results were found: SP142 showed lower levels of PD-L1 expression than 22C3 and 28-8 at 1% cut-off (Figures 2(a)–2(c)), and all assays showed good agreement at the cut-off of 50% (Figures 2(d)–2(f)). Venn diagrams show the positive (Figures 2(b) and 2(e)) and negative agreement (Figures 2(c) and 2(f)) at cut-offs of 1% (Figures 2(b) and 2(c)) and 50% (Figures 2(e) and 2(f)).

### 3.4. Correlation between PD-L1 Expression and Clinical Characteristics

We further investigated the correlation between PD-L1 expression and clinical characteristics to identify possible predictors for PD-L1 expression. PD-L1 expression was associated with N stage but not T and M stages (Table 4). Among the patients with EGFR mutations, 51.0% and 8.2% had PD − L1 > 1% and >50%, respectively, which were lower than PD-L1 levels (PD − L1 > 1%: 63.6%, and PD − L1 > 50%: 16.4%) of patients without EGFR mutation, although this was not statistically significant (*p* = 0.264). However, ALK alterations were not associated with the PD-L1 expression possibly due to the low frequency of patients with ALK alterations. Of note, PD-L1 expression, assessed with 22C3, was significantly associated with PD-L1 expression by Dako28-8 and SP142. Similarly, PD-L1 expressions measured using Dako28-8 and SP142 were significantly associated with the N stage, but not the T and M stages (Supplementary Table S1, S2).

## 4. Discussion

In the current study, we compared the concordance and interchangeability of three different assays/platforms, 22C3, 28-8, and SP142, in assessing PD-L1 expression. Although significantly associated with each other, 22C3 and 28-8 were more compatible than SP142, which showed lower sensitivity to PD-L1 detection. In addition, we found that the PD-L1 expression was significantly associated with advanced N stage but not with the T and M stages.

Although our findings are consistent with previous reports [19, 20] which support high concordance among 22C2 and 28-8 and suggest the interchangeability of both, this should be validated in prospective studies to demonstrate the predictive potential. In a retrospective study of 40 NSCLC patients undergoing nivolumab treatment, the 28-8, 22C3, and SP263 PD-L1 IHC assays showed equivalent predictive performance, whereas the SP142 assay showed lower predictive performance [23]. Prospective studies are needed to validate these findings; however, recent studies usually use PD-L1 as a selection factor or a stratification factor. Studies only use one specific assay, depending on the ICI investigated, for example, 22C3 for pembrolizumab and SP142 for atezolizumab, based on previous successful trials or findings.

In a systemic review of previous studies, none of the FDA-approved in vitro diagnostic devices (IVD) achieved ≥90% sensitivity and specificity for both 1% and 50% TPS cut-offs [24], which was consistent with our findings in the current study. Although the overall agreement for 22C3 and 28-8 was 82.2% and 91.6% for both 1% and 50% TPS cut-offs, respectively, the sensitivity and specificity did not exceed 90%.

Some previous studies using laboratory-derived assays (LDAs) and concordance among LDAs were considered variable [21]. Generally, high concordance was observed among 28-8, 22C3, and SP263 when assessing PD-L1 expression on TCs but not for assessment of PD-L1 expression on ICs [21]. This may be associated with poor interobserver reproducibility for ICs as reported in the Blueprint project [19].

Intraobserver and interobserver reproducibility for PD-L1 IHC is another important issue. In a study designed to test the reproducibility of the assessment of PD-L1 expression (PD-L1 22C3) in NSCLC tissue samples by 10 pathologists, the overall percent agreement (OPA) was approximately 90% and 80% for intraobserver and interobserver reproducibility, respectively, indicating that pathologists reported good reproducibility [25]. However, in the Blueprint project, they found very strong reliability among pathologists in TC PD-L1 scoring with all assays; in contrast, poor reliability was found in IC PD-L1 scoring [19].

In the current study, we found that PD-L1 expression was associated with a higher N stage, which is consistent with one report of 1000 resected lung cancers in Korea which showed that PD-L1 expression in adenocarcinoma was associated with a higher N stage, solid histologic pattern, EGFR wild type, and ALK mutation [26]. Interestingly, PD-L1 expression was associated with M0 rather than M1 stage (*p* = 0.049), and a similar trend was found in our series. In this report, stage III patients had high levels of PD-L1 expression followed by stages II and I and stage IV, which is consistent with our findings. Therefore, locally advanced lung cancer (higher N stage, M0, stage III) may have higher changes in PD-L1 expression than metastatic lung cancer (M1 stage, stage IV); however, the mechanism of tumor biology is unclear. In terms of genetic alterations, PD-L1 expression was associated with EGFR wild type and ALK mutations [26, 27]. Similar trends were found in our series but did not reach statistical significance as limited cases are included in our study.

In conclusion, Dako22C3 and Dako28-8 showed comparable results. Future prospective studies are needed to validate the findings. Clinical features, such as N stage, may be a good predictor for PD-L1 expression.

## Figures and Tables

**Table 1:** Baseline characteristics.

Characteristics	*N* (%)
Age, median (range in years)	65 (28-94)
≤65	73 (50.7%)
>65	71 (49.3%)
Gender	
Male	92 (63.9%)
Female	52 (36.1%)
Histology	
Adenocarcinoma	101 (70.1%)
Squamous cell carcinoma	19 (13.2%)
Adenosquamous carcinoma	5 (3.5%)
NSCLC	19 (13.2%)
T (*n* = 135)	
1	9 (6.7%)
2	25 (18.5%)
3	22 (16.3%)
4	79 (58.5%)
N	
0	13 (9.0%)
1	11 (7.6%)
2	30 (20.8%)
3	90 (62.5%)
M	
0	24 (16.7%)
1	120 (83.3%)
Stage	
I/II	6 (4.2%)
III	18 (12.5%)
IV	120 (83.3%)
EGFR (*n* = 124)	
Mutation	59 (52.4%)
Wild type	65 (47.6%)
ALK (*n* = 121)	
Positive	6 (5.0%)
Negative	115 (95.0%)
PD-L1 (Dako22C3) (*n* = 127)	
<1%	53 (41.7%)
1-49%	56 (44.1%)
≥50%	18 (14.2%)
PD-L1 (SP142 TC/IC) (*n* = 132)	
<1%/<1%	85 (64.4%)
Intermediate	33 (25.0%)
≥50%/>10%	14 (10.6%)
PD-L1 (Dako28-8) (*n* = 110)	
<1%	54 (49.1%)
1-49%	42 (38.2%)
≥50%	14 (12.7%)

NSCLC: non-small cell lung cancer; TC: tumor cells; IC: immune cells.

**Table 2:** Overall agreement between assays (>1%).

Reference	Comparison	*N* of comparison	TP	FN	FP	TN	Sensitivity	Specificity	Agreement	*κ* ^∗^
Dako22C3	SP142^∗^	115	29	38	5	43	43.3%	89.6%	62.6%	0.251
	Dako28-8	107	47	12	7	41	79.7%	85.4%	82.2%	0.645
SP142^∗^	Dako22C3	115	29	5	38	43	85.3%	53.1%	62.6%	0.299
	Dako28-8	109	21	7	34	47	75.0%	58.0%	62.4%	0.251
Dako28-8	Dako22C3	107	47	7	12	41	87.0%	77.4%	82.2%	0.645
	SP142^∗^	109	21	34	7	47	38.2%	87.0%	62.4%	0.299

TP: true positive; TN: true negative; FP: false positive; FN: false negative; TC: tumor cells; IC: immune cells. SP142 was scored by TC/IC > 1%/1%. ^∗^Cohen's kappa coefficient (*κ*).

**Table 3:** Overall agreement between assays (>50%).

Reference	Comparison	*N* of comparison	TP	FN	FP	TN	Sensitivity	Specificity	Agreement	*κ* ^∗^
Dako22C3	SP142^∗^	115	8	8	2	97	50.0%	98.0%	91.3%	0.569
	Dako28-8	107	8	3	6	90	72.7%	93.8%	91.6%	0.593
SP142^∗^	Dako22C3	115	8	2	8	97	80.0%	92.4%	91.3%	0.569
	Dako28-8	109	7	2	7	94	77.8%	93.1%	91.8%	0.565
Dako28-8	Dako22C3	107	8	6	3	90	57.1%	96.8%	91.6%	0.593
	SP142^∗^	109	7	7	2	94	50.0%	97.9%	91.8%	0.565

TP: true positive; TN: true negative; FP: false positive; FN: false negative; TC: tumor cells; IC: immune cells. SP142 was scored by TC/IC > 50%/10%. ^∗^Cohen's kappa coefficient (*κ*).

**Table 4:** Patients' characteristics and association with PD-L1 (Dako22C3).

Characteristics	PD-L1 (Dako22C3)			*p* value
	<1% (*N* = 53)	1-49% (*N* = 56)	≥50% (*N* = 18)	
Age, median (years)	65 (30-93)	66 (43-94)	66 (44-81)	
≤65	28 (43.8%)	27 (42.2%)	9 (14.1%)	0.890
>65	25 (39.7%)	29 (46.0%)	9 (14.3%)	
Gender				
Male	30 (36.1%)	41 (49.4%)	12 (14.5%)	0.189
Female	23 (52.3%)	15 (34.1%)	6 (13.6%)	
Histology				
Adenocarcinoma	41 (46.6%)	36 (40.9%)	11 (12.5%)	0.759
SqCC	4 (25%)	9 (56.3%)	3 (18.8%)	
Adenosquamous carcinoma	2 (40.0%)	2 (40.0%)	1 (20.0%)	
NSCLC	6 (33.3%)	9 (50.0%)	3 (16.7%)	
T				
1	5 (62.5%)	3 (37.5%)	0 (0.0%)	0.566
2	10 (47.6%)	7 (33.3%)	4 (19.0%)	
3	9 (45.0%)	10 (50.0%)	1 (5.0%)	
4	28 (39.4%)	31 (43.7%)	12 (16.9%)	
N				
0	5 (50.0%)	4 (40.0%)	1 (10.0%)	0.031
1	7 (77.8%)	2 (22.2%)	0 (0.0%)	
2	15 (62.5%)	6 (25.0%)	3 (12.5%)	
3	26 (31.0%)	44 (52.4%)	14 (16.7%)	
M				
0	10 (45.5%)	7 (31.8%)	5 (22.7%)	0.306
1	43 (41.0%)	49 (46.7%)	13 (12.4%)	
Stage				
I/II	3 (60.0%)	1 (20.0%)	1 (20.0%)	0.568
III	7 (41.2%)	6 (35.3%)	4 (23.5%)	
IV	43 (41.0%)	49 (46.7%)	13 (12.4%)	
EGFR				
Mutation	24 (49.0%)	21 (42.9%)	4 (8.2%)	0.264
Wild type	22 (36.1%)	29 (47.5%)	10 (16.4%)	
ALK				
Positive	1 (16.7%)	4 (66.7%)	1 (16.7%)	0.412
Negative	45 (44.1%)	44 (43.1%)	13 (12.7%)	
PD-L1 (SP142 TC/IC)				
<1%/<1%	43 (53.1%)	35 (43.2%)	3 (3.7%)	<0.001
Intermediate	4 (16.7%)	15 (62.5%)	5 (20.8%)	
≥50%/>10%	1 (10.0%)	1 (10.0%)	8 (80.0%)	
PD-L1 (Dako28-8)				
<1%	41 (77.4%)	11 (20.8%)	1 (1.9%)	<0.001
1-49%	7 (17.5%)	31 (77.5%)	2 (5.0%)	
≥50%	0 (0.0%)	6 (42.9%)	8 (57.1%)	

Figures are numbers with percentages in parentheses, unless otherwise stated. The chi-squared test of independence: categorical variable. NSCLC: non-small cell lung cancer; TC: tumor cells; IC: immune cells.

## Data Availability

The datasets generated and analyzed during the current study are not publicly available due to IRB regulation but are available from the corresponding author on reasonable request.

## Supplementary Materials

Supplementary Materials Supplementary Table S1: patients' characteristics and association with PD-L1 (Dako28-8). Supplementary Table S2: patients' characteristics and association with PD-L1 (SP142).
