# Supplementary material for: PD-L1 Immunohistochemistry Comparability and Their Correlation with Clinical Characteristics in NSCLC
Source: Anal Cell Pathol (Amst). 2020 Nov 2;2020:3286139. doi: 10.1155/2020/3286139 (PMC7655253; doi:10.1155/2020/3286139)
Supplement: Supplementary Materials — Supplementary Table S1: patients' characteristics and association with PD-L1 (Dako28-8). Supplementary Table S2: patients' characteristics and association with PD-L1 (SP142). [file 3286139.f1.docx]

Supplementary Table S1. Patients’ Characteristics and Association with PD-L1(Dako28-8)

| Characteristics | | PD-L1(Dako28-8) | | | | | | *P*  value | |
| --- | --- | --- | --- | --- | --- | --- | --- | --- | --- |
|  |  | < 1%  (N=54) | | 1-49%  (N=42) | | > 50%  (N=14) | |  |  |
| Age, median (IQR) | | 65(33-94) | | 68.5(42-91) | | 66(46-81) | |  | |
| ≦65 | | 28(53.8%) | | 17(32.7%) | | 7(13.5%) | | 0.529 | |
| >65 | | 26(44.8%) | | 25(43.1%) | | 7(12.1%) | |  | |
| Gender | |  | |  | |  | |  | |
| Male | | 35(50.7%) | | 24(34.8%) | | 10(14.5%) | | 0.573 | |
| Female | | 19(46.3%) | | 18(90.2%) | | 4(9.8%) | |  | |
| Histology |  | |  | |  | |  | |  |
| Adenocarcinoma | | 39(51.3%) | | 27(35.5%) | | 10(13.2%) | | 0.744 | |
| SqCC | | 5(35.7%) | | 6(42.9%) | | 3(21.4%) | |  | |
| Adenosquamous | | 2(40.0%) | | 3(60.0%) | | 0(0%) | |  | |
| NSCLC | | 8(53.3%) | | 6(40.0%) | | 1(6.7%) | |  | |
| T | |  | |  | |  | |  | |
| 1 | | 6(75.0%) | | 2(25.0%) | | 0(0%) | | 0.426 | |
| 2 | | 11(57.9%) | | 5(26.3%) | | 3(15.8%) | |  | |
| 3 | | 9(60.0%) | | 4(26.4%) | | 2(13.3%) | |  | |
| 4 | | 26(41.3%) | | 28(44.4%) | | 9(14.3%) | |  | |
| N | |  | |  | |  | |  | |
| 0 | | 7(70.0%) | | 3(30.0%) | | 0(0%) | | 0.049 | |
| 1 | | 7(87.5%) | | 1(12.5%) | | 0(0%) | |  | |
| 2 | | 13(59.1%) | | 5(22.7%) | | 4(18.2%) | |  | |
| 3 | | 27(38.6%) | | 33(47.1%) | | 10(14.3%) | |  | |
| M | |  | |  | |  | |  | |
| 0 | | 10(52.6%) | | 7(36.8%) | | 2(10.5%) | | 0.925 | |
| 1 | | 44(48.4%) | | 35(38.5%) | | 12(13.2%) | |  | |
| Stage | |  | |  | |  | |  | |
| I/II | | 3(60.0%) | | 2(40.0%) | | 0(0%) | | 0.933 | |
| III | | 7(50.0%) | | 5(35.7%) | | 2(14.3%) | |  | |
| IV | | 44(48.4%) | | 35(38.5%) | | 12(13.2%) | |  | |
| EGFR | |  | |  | |  | |  | |
| Mutation | | 26(54.2%) | | 19(39.6%) | | 3(6.3%) | | 0.259 | |
| Wild type | | 23(48.9%) | | 16(34.0%) | | 8(17.0%) | |  | |
| ALK | |  | |  | |  | |  | |
| Positive | | 1(25.0%) | | 2(50.0%) | | 1(25.0%) | | 0.497 | |
| Negative | | 48(52.2%) | | 34(37.0%) | | 10(10.9%) | |  | |
| PD-L1 (Dako22c3) | |  | |  | |  | |  | |
| <1% | | 41(85.4%) | | 7(14.6%) | | 0(0.0%) | | <0.001 | |
| 1-49% | | 11(22.9%) | | 31(64.6%) | | 6(12.5%) | |  | |
| >50% | | 1(9.1%) | | 2(18.2%) | | 8(72.7%) | |  | |
| PD-L1 (SP142) | |  | |  | |  | |  | |
| <1% and <1% | | 47(58.0%) | | 30(37.0%) | | 4(4.9%) | | <0.001 | |
| 1-49% and 1-9% | | 6(31.6%) | | 10(52.6%) | | 3(15.8%) | |  | |
| >50% or >10% | | 1(11.1%) | | 1(11.1%) | | 7(77.8%) | |  | |
| Figures are numbers with percentages in parentheses, unless otherwise stated.  The Chi-Squared test of independence: categorical variable  NSCLC, non-small cell lung cancer; TC, tumor cells; IC, immune cells. | | | | | | | | | |

Supplementary Table S2. Patients’ Characteristics and Association with PD-L1(SP142)

| Characteristics | PD-L1(SP142 TC/IC) | | | *P*  value |
| --- | --- | --- | --- | --- |
|  | < 1% and <1%  (N=85) | Intermediate  (N=33) | > 50% or > 10%  (N=14) |  |
| Age, median (IQR) | 69(41-94) | 61(42-89) | 64.5(33-83) |  |
| ≦65 | 36(54.5%) | 23(34.8%) | 7(10.6%) | 0.029 |
| >65 | 49(74.2%) | 10(15.2%) | 7(10.6%) |  |
| Gender |  |  |  |  |
| Male | 51(60.7%) | 22(26.2%) | 11(13.1%) | 0.374 |
| Female | 34(70.8%) | 11(22.9%) | 3(6.3%) |  |
| Histology |  |  |  |  |
| Adenocarcinoma | 59(64.8%) | 26(28.6%) | 6(6.6%) | 0.3 |
| SqCC | 11(61.1%) | 4(22.2%) | 3(16.7%) |  |
| Adenosquamous | 4(80.0%) | 0(0%) | 1(20.0%) |  |
| NSCLC | 11(61.1%) | 3(16.7%) | 4(22.2%) |  |
| T |  |  |  |  |
| 1 | 5(55.6%) | 3(33.3%) | 1(11.1%) | 0.611 |
| 2 | 15(65.2%) | 4(17.4%) | 4(17.4%) |  |
| 3 | 17(81.0%) | 3(14.3%) | 1(4.8%) |  |
| 4 | 45(63.4%) | 19(26.8%) | 7(9.9%) |  |
| N |  |  |  |  |
| 0 | 7(53.8%) | 4(30.8%) | 2(15.4%) | 0.871 |
| 1 | 7(70.0%) | 2(20.0%) | 1(10.0%) |  |
| 2 | 15(55.6%) | 8(29.6%) | 4(14.8%) |  |
| 3 | 56(68.3%) | 19(23.2%) | 7(8.5%) |  |
| M |  |  |  |  |
| 0 | 14(58.3%) | 5(20.8%) | 5(20.8%) | 0.196 |
| 1 | 71(65.7%) | 28(25.9%) | 9(8.3%) |  |
| Stage |  |  |  |  |
| I/II | 3(50.0%) | 1(16.7%) | 2(33.3%) | 0.333 |
| III | 11(61.1%) | 4(22.2%) | 3(16.7%) |  |
| IV | 71(65.7%) | 28(25.9%) | 9(8.3%) |  |
| EGFR |  |  |  |  |
| Mutation | 39(70.9%) | 12(21.8%) | 4(7.3%) | 0.342 |
| Wild type | 33(57.9%) | 17(29.8%) | 7(12.3%) |  |
| ALK |  |  |  |  |
| Positive | 3(50.01%) | 3(50.0%) | 0(0.0%) | 0.312 |
| Negative | 71(65.7%) | 26(24.1%) | 11(10.2%) |  |
| PD-L1 (Dako22C3) |  |  |  |  |
| <1% | 43(89.6%) | 4(8.3%) | 1(2.1%) | <0.001 |
| 1-49% | 35(68.6%) | 15(29.4%) | 1(2.0%) |  |
| >50% | 3(18.8%) | 5(31.3%) | 8(50.0%) |  |
| PD-L1 (Dako28-8) |  |  |  |  |
| <1% | 47(87.0%) | 6(11.1%) | 1(1.9%) | <0.001 |
| 1-49% | 30(73.2%) | 10(24.4%) | 1(2.4%) |  |
| >50% | 4(28.6%) | 3(21.4%) | 7(50.0%) |  |

Figures are numbers with percentages in parentheses, unless otherwise stated.

The Chi-Squared test of independence: categorical variable

NSCLC, non-small cell lung cancer; TC, tumor cells; IC, immune cells.
